# Review: The Lacrimal Gland and Its Role in Dry Eye

**DOI:** 10.1155/2016/7542929

**Published:** 2016-03-02

**Authors:** Christopher D. Conrady, Zachary P. Joos, Bhupendra C. K. Patel

**Affiliations:** Department of Ophthalmology, Division of Ophthalmic Plastic and Reconstructive Surgery, John A. Moran Eye Center, University of Utah, Salt Lake City, UT, USA

## Abstract

The human tear film is a 3-layered coating of the surface of the eye and a loss, or reduction, in any layer of this film may result in a syndrome of blurry vision and burning pain of the eyes known as dry eye. The lacrimal gland and accessory glands provide multiple components to the tear film, most notably the aqueous. Dysfunction of these glands results in the loss of aqueous and other products required in ocular surface maintenance and health resulting in dry eye and the potential for significant surface pathology. In this paper, we have reviewed products of the lacrimal gland, diseases known to affect the gland, and historical and emerging dry eye therapies targeting lacrimal gland dysfunction.

## 1. Introduction

The human tear film coats the anterior surface of the eye and is composed of three distinct layers: an inner mucin coating, a middle aqueous component, and a lipid overlay. Traditionally, the mucin layer was felt to be derived from goblet cells of the conjunctiva, the aqueous component from the lacrimal gland, and the lipid layer from the meibomian glands [1–3]. Recent advancements in proteomics have slightly altered this view of the tear film by identifying mucin as a product of the goblet cells but the lacrimal gland as well [4]. The 3-layered tear film inhibits ocular surface invasion by pathogens, provides an air-tissue interface for gas exchange, and supplies essential nutrients and metabolites to maintain a transparent and avascular cornea. The lacrimal gland contributes multiple components to the tear film and has been the center of much research including multiple products now under clinical trials. In the this paper we review the anatomy, physiology, and normal products of the lacrimal gland in regard to their role in dry eye diseases. We have also reviewed specific causes of lacrimal gland pathology such as aging, smoking, autoimmune diseases, and infections. Finally, the historical and emerging treatments for dry eye related to lacrimal gland dysfunction with an emphasis on surgical approaches are detailed within.

## 2. Anatomy, Physiology, Innervation, and Histology

A proper review of the anatomy of the lacrimal gland and accessory lacrimal tissues is important for understanding the pathophysiology of dry eye syndrome and secondary causes of dry eye.

### 2.1. Anatomy, Blood Supply, Innervation

**Figure 1:**
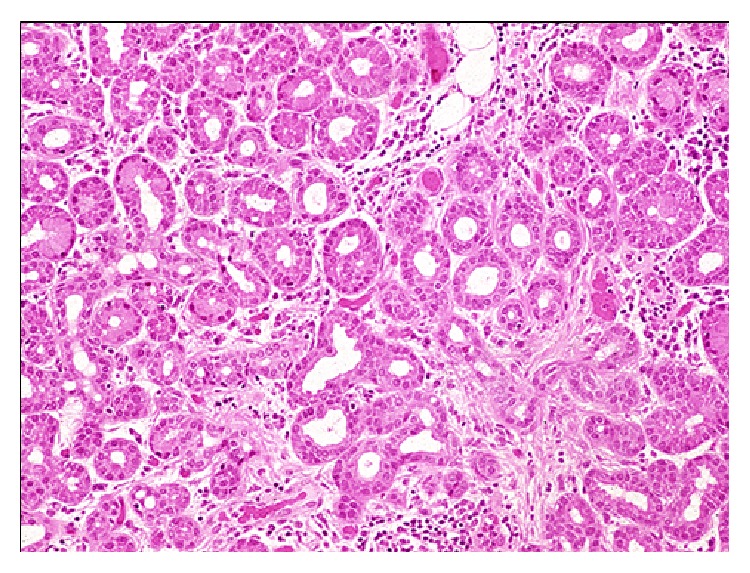
Lacrimal gland histopathology. H&E staining of a normal lacrimal gland. The gland is composed of lobules separated by loose connective tissue. The lobules are composed of multiple acini lined by columnar secretory cells.

**Figure 2:**
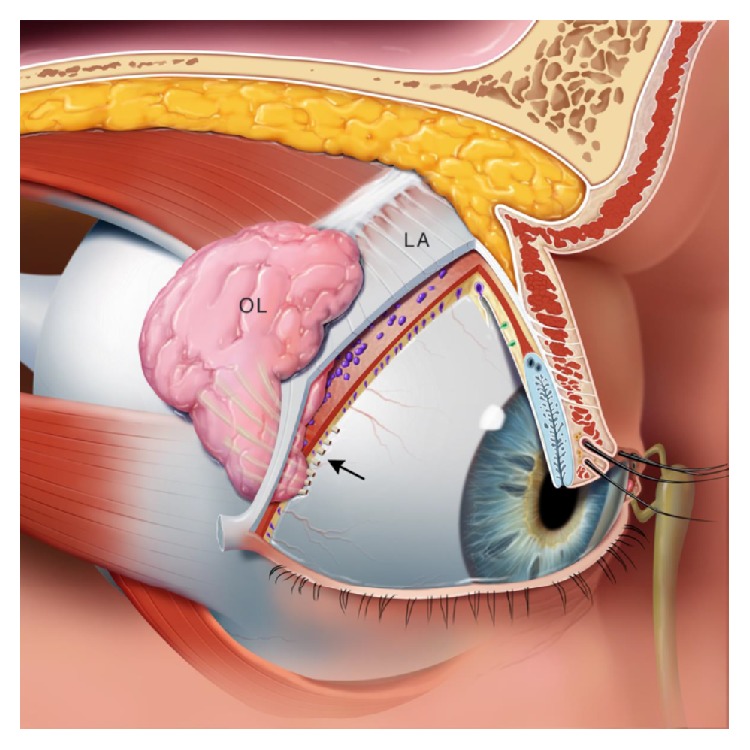
Oblique view of the right orbit. Oblique view of the right orbit showing the main lacrimal gland divided into the orbital lobe (OL) and palpebral lobe by the lateral horn of the levator aponeurosis (LA). Note the excretory ducts coursing through the palpebral lobe and draining into the superior conjunctival fornix (arrow).

**Figure 3:**
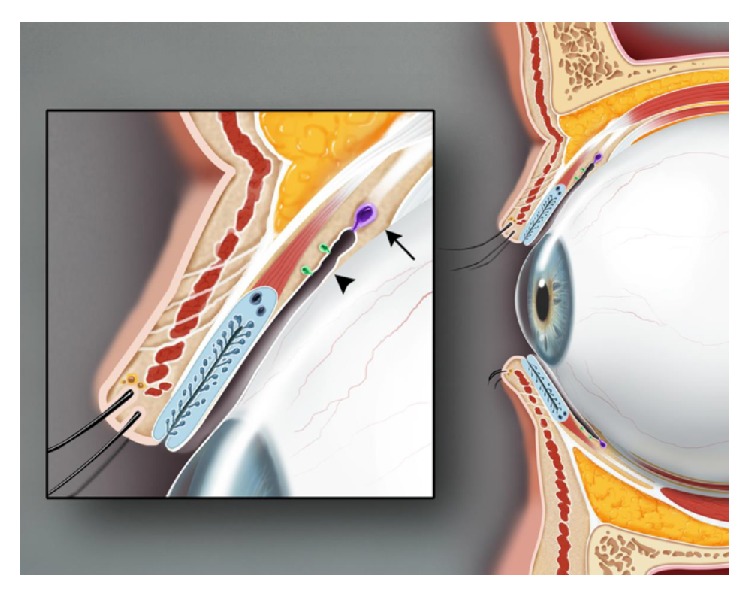
Sagittal view of the upper and lower eyelids. The glands of Krause (arrow) are located in the superior conjunctival fornix. The glands of Wolfring (arrowhead) are found at the nonmarginal border of the tarsal plate.

Embryologically, the main lacrimal gland develops from an outpouching of the conjunctiva. The accessory lacrimal glands develop slightly later than the main lacrimal gland [5]. The main lacrimal gland is situated superotemporally in the orbit within the lacrimal fossa of the frontal bone. Grossly, the gland is a pinkish-gray structure composed of small lobules intermixed with connective tissue septations and lacks a true capsule (Figure 1). Its appearance may be mistaken for preaponeurotic fat. The gland is divided into two lobes, the orbital and palpebral lobes, by the lateral horn of the levator aponeurosis. Although divided, the division is incomplete due to a posterior wall of parenchyma between the lobes [5]. The gland is bound anteriorly by the orbital septum and the preaponeurotic fat pad, posteriorly by orbital fat, medially by the intermuscular membrane between the superior and lateral recti, and laterally by bone (Figure 2). The size of the main lacrimal gland is somewhat variable with the orbital lobe being the larger of the two. The gland averages approximately 20 mm long and 12 mm wide with the orbital and palpebral lobes having a thickness of 5 mm and 3 mm, respectively [6, 7]. The palpebral lobe lies beneath the levator aponeurosis in the subaponeurotic Jones' space [5]. The gland is supported by conjunctiva, intermuscular membranes, its facial attachments to Whitnall's ligament, and the levator horn (Figures 2 and 3).

The lacrimal gland is an exocrine gland similar to the mammary gland and salivary gland [7]. The gland is composed of lobules separated by loose connective tissue (Figure 1). Acini are lined with columnar secretory cells, which have been shown to secrete mucopolysaccharides, implying that the gland is a modified mucus gland [5]. Each lacrimal gland lobule consists of many acini and intralobular ducts that drain into approximately 8–12 excretory ducts or tubules. The ducts of both the orbital and palpebral lobes drain into the superotemporal conjunctival fornix, approximately 5 mm superior the lateral tarsal border [8]. The ducts of the orbital lobe pass through the parenchyma of the palpebral lobe making the proximal secretory ducts susceptible to damage distally [5, 7, 8].

The arterial blood supply to the lacrimal gland comes from the lacrimal branch of the ophthalmic artery, a branch of the infraorbital artery, and occasionally from a branch of the recurrent meningeal artery. The lacrimal artery passes through the gland to feed the upper and lower eyelids. The lacrimal vein follows the course of the artery and drains into the superior ophthalmic vein.

The gland is innervated by both myelinated and unmyelinated fibers arising from the trigeminal nerve, the facial nerve, and sympathetic innervation from the superior cervical ganglion [5]. Stimulation of the ocular surface activates tear production from the main lacrimal gland (reflex tearing). The lacrimal nerve is a sensory branch of the ophthalmic trigeminal nerve (V_1_), which provides the sensory (afferent) pathway. This lacrimal nerve travels in the superotemporal orbit and enters the gland with the major vessels. This nerve courses through the gland to innervate superficial eyelid structures. Sympathetic nerves travel with the lacrimal artery along with parasympathetics in the zygomatic nerve [5].

The efferent pathway originates with parasympathetic fibers from the superior salivary nucleus of the pons, which exit the brain stem with the facial nerve. Lacrimal fibers depart from the facial nerve as the greater superficial petrosal nerve and travel to the sphenopalatine ganglion to join the zygomatic nerve. The zygomatic nerve enters the orbit 5 mm posterior to the anterior limit of the inferior orbital fissure. Prior to dividing into the zygomaticotemporal and zygomaticofacial branches, the zygomatic nerve may give off a lacrimal branch, which may anastomose with a branch of the lacrimal nerve or travel independently along the periorbita [5]. It is unclear if the anastomosis between the zygomaticotemporal and lacrimal nerves is uniformly present [8]. The role of the sympathetic nervous system is thought to stimulate basal tear secretion, but its role in lacrimation is not well understood. Lacrimal gland hyposecretion is seen in syndromes of central autonomic dysfunction, such as Riley-Day syndrome [9].

There are approximately 20 glands of Krause located in the superior conjunctival fornix and approximately half as many in the inferior fornix. The glands of Wolfring are found along the nonmarginal border of the both tarsal plates (Figures 2 and 3) [5]. Accessory lacrimal glands may also be found in the caruncle and in the plica semilunaris. The accessory glands account for approximately 10% of the total lacrimal secretory mass [8]. Although the accessory lacrimal glands of Krause and Wolfring are structurally and histologically similar to the main lacrimal gland and may develop identical types of metaplasia, they differ in their innervation [7]. Although heavily innervated, the accessory lacrimal glands lack parasympathetic innervation [5], and most of the innervation is unidentified [8]. Jones states that the main lacrimal gland is responsible only for reflex tearing and the accessory glands of Kraus and Wolfring, providing basal tear secretion [10]. This distinction has been debated. The volume of tears secreted from these glands is unclear. Studies show mixed results whether or not the accessory glands are able to provide adequate tear volume to prevent keratoconjunctivitis sicca [7].

### 2.2. Pathology

Noted age-related changes of the lacrimal gland include atrophy of the glandular parenchyma, increased interstitial connective tissue, increased fat content within glandular tissue and epithelial secretory cells, and increased lymphocyte content within the gland including plasma cells [11–13]. The incidence and uniformity of these changes have not been agreed upon, as many reports note conflicting data.

Obata et al. found that lobular fibrosis, lobular atrophy, diffuse fibrosis, diffuse atrophy, periductal fibrosis, lymphocytic foci, and fatty infiltration were found significantly more often in orbital lobes, whereas interlobular ductal dilatation was observed more frequently in palpebral lobes [11]. It is unknown if structural and functional differences exist between the orbital and palpebral lobes or if these differences represent continuum of changes versus distinct pathophysiologic changes.

An autopsy study of lacrimal glands by Roen and colleagues found that 75% of glands studied showed microscopic abnormalities [12]. The most common abnormal findings included chronic inflammation and periductular fibrosis. Approximately 52% and 74% of patients over the age of 50 showed signs of periductular fibrosis and ductal abnormalities, respectively [12]. The authors also observed massive ductular ectasia extending into lobules. The combination of periductular fibrosis, inflammation, and dilated, inspissated ducts may lead to retention of tears within the lacrimal gland and contribute to age-related dry eye [12]. Another study by Obata et al. found a statistically significant difference in incidence of diffuse fibrosis, atrophy, and periductal fibrosis of the lacrimal gland in postmenopausal women compared to men [11]. Glands in which acinar atrophy is apparent show a lack of lysozyme immunoreactivity and are probably related to a decrease of tear proteins as a consequence of aging [7]. Atrophy of acinar elements may result in fibrosis, but in certain conditions, such as chronic graft-versus-host disease, stromal fibroblasts are actively involved in the pathogenic process of periacinar fibrosis [14]. The health of the conjunctival epithelium is essential for normal lacrimal gland function. Stenosis or obstruction of flow of the excretory ducts in the superior conjunctival fornix may cause cystic dilatation of the interlobular ducts in the palpebral lobe. Damage to the excretory ducts in the superior conjunctiva may occur with severe ocular surface diseases with keratinization such as Stevens-Johnson syndrome and ocular cicatricial pemphigoid, or iatrogenically after surgery, which may damage the orifices of the excretory ducts thereby reducing the volume of aqueous bathing the ocular surface [7, 15].

### 2.3. Contributions of the Lacrimal Gland to Ocular Surface Health

As previously suggested, the components of the tear film produced by the lacrimal gland are critical in several processes related to ocular surface health. The first is in protection of the ocular surface from invading pathogens with a local population of IgA-secreting plasma cells that reside within the lacrimal gland itself. While tear firm contains other immunoglobulins, secretory IgA is the predominant antibody and is the only immunoglobulin whose concentration significantly increases during infection, suggesting its critical role in host defense of the ocular surface [16]. The ability of the lacrimal gland to specifically select for IgA secreting plasma cells is not well understood but likely resides in the recruitment and proliferation of a specific subset of helper T cells. These T cells are recruited by an IL-2-like peptide known as lacrimal gland-derived lymphocyte proliferation potentiating factor [17, 18]. These T cells then recruit and promote B cell differentiation into IgA-secreting plasma cells. Once produced by plasma cells, dimeric IgA is translocated into the tear film by a cell surface antibody receptor to inhibit pathogen adherence to the host surface as seen at other mucosal sites [19]. The production of this translocation receptor is exquisitely sensitive to endocrine and nervous and immune system regulation [20, 21]. Consequently, the host invests significant energy into the production and secretion of IgA into the tear film to reduce ocular surface susceptibility.

The lacrimal gland also secretes several bacteria (i.e., secretory phospholipase A2, an effective antistaphylococcal enzyme among others [22]) and fungicidal agents such as lysozyme, peroxidase, tear-specific pre-albumin, psoriasin, and lactoferrin into the tear film [2, 23]. These substances greatly reduce susceptibility of the ocular surface due to cytotoxicity to invading pathogens. While it is still controversial, the lacrimal gland may also be an additional source of soluble mucin production, which acts to clear debris and hold fluid on the surface of the eye [24–26]. This glycoprotein also serves as an infectious deterrent by acting as a decoy receptor for invading pathogens [27]. As such, these cytotoxic agents, mucin, and IgA transform a susceptible, warm, moist, nutrient rich epithelial surface into an inhospitable environment unlike other colonized mucosal surfaces.

The second major contribution of the lacrimal gland is in the aqueous produced by acinar cells that add significant volume to the tear film. The fluid is transported from the interstitial space into the lumen of the gland by way of osmosis and released onto the ocular surface [2]. The addition of high volumes of water from the gland helps to keep the ocular surface moist, maintain an important component of light refraction in the air-water-corneal interfaces, and dilute proteins within the tears to keep them solubilized. Water is also transported in conjunction with other important electrolytes required in cellular processes and has been extensively reviewed elsewhere [2]. With the addition of lipocalin and lipids from the meibomian gland, tears become a highly viscous, low surface tension solution critical in tear film stability and health of the ocular surface [28]. As such, water serves to dilute substances in the tear film and maintain an interface critical for normal visual acuity.

The lacrimal gland is also responsible for producing several other proteins and products necessary in growth and maintenance of host tissue found in the tear film. Several of these proteins are growth factors. They include epidermal, fibroblast, hepatocyte, keratinocyte, and transforming growth factor-*β*. While the defined role of each in corneal regeneration is unclear, these factors promote proliferation and migration of epithelial cells following disruption of the corneal surface and maintain an avascular cornea necessary for transparency of the tissue [29–34]. If these factors decline or are replaced for others, neovascularization of the cornea ensues [35, 36].

Retinol, a vitamin A derivative, is also secreted by the lacrimal gland. Retinol is required in maintenance of goblet cells within the conjunctiva and controls corneal epithelial desquamation, keratinization, and metaplasia [37–39]. Vitamin A is also a positive feedback molecule as its deficiency results in a decrease in flow rate of lacrimal gland fluid in rabbits [40]. In humans, vitamin A deficiency can result in corneal ulcers, melt, and even perforation [41]. This loss of corneal integrity is felt to be the result of an increased risk of infection, decreased tear film, alterations in corneal wound healing, and changes in leukocyte function [42]. Consequently, the ocular surface role of secreted vitamin A from the lacrimal gland is multifactorial. The previously mentioned products of the lacrimal gland are only a select few of the known proteins in the tear film and there are likely several unidentified proteins at this point in time.

In summary, the lacrimal gland secretes a complex aqueous milieu rich in antibodies, cytotoxic agents, and growth factors onto the ocular surface to protect the cornea from desiccation, infection, and vascularization while promoting wound healing and transparency.

### 2.4. Disease of the Lacrimal Gland

Dysfunction of the lacrimal gland may result from inflammation, aging, radiation, or infection. The end result of many of these pathologies rests in insufficient tear production and changes in osmolality and increased osmotic stress of the ocular surface [43]. This results in increased susceptibility of the ocular surface that we hypothesize is due to the loss of the previously mentioned antimicrobial tear film products [44]. Unfortunately, in inflammatory dry eye, this is further exacerbated by relatively high concentrations of proteins within the tears that induce apoptosis of surface epithelium and a vicious, self-perpetuated cycle of increased expression of proinflammatory cytokines from the ocular surface [45, 46]. The proinflammatory state further worsens dry eye by leading to apoptosis and decreased mucin production from conjunctival goblet cells [47, 48]. Matrix metalloproteinases (MMPs), a family of proteins required in wound healing and degradation of extracellular matrix, are one such proinflammatory product highly expressed in dry eye conditions and known to cause epithelial barrier dysfunction [45, 49]. As such, tests such as InflammaDry by Rapid Pathogen Screening have been developed to evaluate tear concentrations of MMPs as surrogates for inflammation in the clinical realm [50].

In the following section, we have focused on specific diseases to highlight the major causes of lacrimal dysfunction, that is, Sjogren's syndrome (SS) as a representative for inflammation (Table 1). Several diseases cause multiple types of pathology making gross categorization difficult.

### 2.5. Aging

Aging takes a toll on the entire body and the lacrimal gland is no different resulting in decreased tear production with increasing age [51]. Progressive acinar atrophy and fibrosis and lymphocytic infiltrates are more common within the lacrimal glands of the elderly [52]. While the exact pathophysiological changes are not well understood, mice lacking a major antioxidant pathway have been shown to have more extensive acinar atrophy and a larger leukocyte infiltrate within the lacrimal gland compared to controls [53]. Furthermore, there is likely some component of autoimmune-driven destruction of the gland with aging as CD4^+^ T cell adoptive transfers from elderly mice into naïve, immunodeficient recipients that results in a reduction of goblet cells and T cell infiltrate into the lacrimal gland. Unfortunately, this study did not correlate pathology of the gland with this immune infiltrate [54]. As such, the exact role of this T cell infiltrate into the lacrimal gland of the elderly is undefined; however, speculation would surmise that this may result in an inflammatory dry eye disease process with lacrimal gland destruction similar to SS. This is supported in part in that the tear film of older mice contains higher concentrations of pro-inflammatory cytokines than younger mice [55]. In humans this is further supported by the upregulation of inflammatory markers with decreased aqueous production in the elderly [56]. In total, lacrimal gland hypofunction in the elderly is likely the result of oxidative damage and an ongoing autoimmune, inflammatory event.

### 2.6. Inflammatory Diseases of the Lacrimal Gland

SS is a systemic, chronic inflammatory state of the exocrine glands predominately seen in women that results in dry eyes and mouth. The initiating environmental factor or pathogen trigger for glandular inflammation defining the disease is unknown. A lymphocytic infiltrate, predominately activated CD4^+^ T cells, is responsible for the enlargement and permanent damage of the exocrine glands resulting in reduced secretions and breakdown of mucosal surfaces [57–59]. In regard to the lacrimal gland itself, imaging studies have shown an accelerated fat deposition within the gland during SS and histopathologic changes such as intralobular fibrosis and a disorganized arrangement of the ducts occurs in even mild cases [60, 61]. Furthermore, inflammation involving the lacrimal ducts likely complicates aqueous outflow but little is known on the subject. The role of each of these changes in the overall reduction in tear production is still debatable as the degree of tissue destruction and lymphocytic infiltrate does not correlate with the level of gland dysfunction [62–64].

Despite extensive research, the exact pathophysiology of the disease remains unclear. What is clear, however, is that the tear film of patients with SS contains an inflammatory proteomic profile compared to normal controls [65]. This presumably results in epithelial decompensation and loss of goblet cells as previously described resulting in severe dry eye. In mouse models, the lacrimal and submandibular glands are the first affected in the disease process, and MMPs and other proinflammatory cytokines are upregulated in tear film [66–68]. To make matters worse, dry conditions trigger significant production of proinflammatory mediators in SS patient's within hours of introduction into the environment suggesting a frailty of the tissue [46]. As the disease progresses, lacrimal gland production wanes necessitating increased ocular lubrication and the addition of topical anti-inflammatories such as cyclosporine [69].

While the mechanism is likely similar to SS with an abnormal immune response, it is worth at least mentioning a fairly new entity, IgG4-related disease, that can cause lacrimal gland dysfunction and is a current, popular topic in the clinical and scientific realm [70, 71]. The disease is characterized by an infiltration of IgG4-producing plasma cells, elevated serum IgG4, and fibrosis and enlargement of multiple organs and was previously known under the eponym Milkulicz's disease [70, 72]. These changes within the lacrimal gland can induce dry eye. Consequently, IgG4-related disease is a known inflammatory disorder of the lacrimal gland but not as well understood as that of SS.

### 2.7. Environmental: Smoking and Video Displays

Smoking and video displays have been implicated in lacrimal gland dysfunction [73, 74]. While the mechanism of gland dysfunction is unclear in both, cytochrome P450s and signals of oxidative damage are upregulated in the lacrimal glands of rats exposed to cigarette smoke [73]. We hypothesize that this likely results in destruction of the gland as seen with an aging lacrimal gland; however, no study has specifically evaluated the underlying pathophysiology. In regard to video displays, lacrimal gland hypofunction and decreased tear production are dependent on the amount of time the monitor is used at work. Unfortunately untested, the authors speculate that proper lacrimal gland function is dependent on number of eyelid blinks [74]. To partially support this, patients with Parkinson's disease have poor blink rates, tear meniscus heights, and dry eye [75]. Whether blink rate and subsequent lacrimal gland hypofunction is a contributor of dry eye in Parkinson's disease is unknown, however. Regrettably, environmental causes of lacrimal gland dysfunction are poorly understood and there are likely many other factors responsible for decreased tear production that have not been identified.

### 2.8. Infectious: HIV

Dry eye is more prevalent in HIV patients than in the general public with a study reporting more than 85% of these patients to have findings consistent with dry eye [76, 77]. A portion of patients clearly show a reduction in tear production [78] and this is hypothesized to be due to a lymphocytic infiltrate similar to SS [79]. This is most evident in those HIV-infected patients who develop diffuse infiltrative lymphocytosis syndrome, a rare entity since the introduction of HAART. In these patients, the salivary and lacrimal glands enlarge with CD8^+^ lymphocytes [79]. As such, lacrimal gland dysfunction during infectious diseases is likely a similar pathophysiological event as that found in SS. Therapeutic options are few as topical cyclosporine suppresses local immunity and is likely a poor choice for this case of inflammatory dry eye.

### 2.9. Radiation

Many head and neck cancers are treated with surgical and/or radiation therapy. While radiation is an effective treatment of rapidly dividing cancerous cells, this therapy has well known toxic effects on local and regional tissues resulting in side effects reviewed extensively elsewhere [80]. Despite the glands of the head being highly differentiated and slowly dividing tissues, they are exquisitely sensitive to radiation that can cause transient and/or permanent dysfunction of the gland [81, 82]. Xerostomia is the most common presentation of glandular dysfunction of the head and neck; however, the lacrimal gland is also affected by radiation [83]. In rabbits, loss of smooth muscle and decreased aqueous secretion occur within 3 days of irradiation of the lacrimal gland and persist beyond thirty days [84]. Unfortunately, the long-term histopathological effects of radiation on the lacrimal gland have been poorly studied in animals and humans. In patients receiving local radiation, lacrimal gland dysfunction results in a dose-dependent increase in severity of dry eye following radiation treatment [85]. Consequently, pathological changes occur within days of radiation therapy inducing both temporary and permanent lacrimal gland dysfunction and resultant dry eye. While dry eyes are an unfortunate side effect, radiation therapy of head, neck, and orbit remains a commonly used treatment modality due to its success in treating such tumors making radiation-induced dry eye an issue for the foreseeable future [80].

### 2.10. Idiopathic

Lastly, there are idiopathic causes of lacrimal gland dysfunction that cannot be linked to any specific cause that may represent subclinical presentations of those previously mentioned above or an altogether undefined entity.

## 3. Historical Treatment of Dry Eye Related to Lacrimal Gland Dysfunction

Regrettably, the treatment of dry eye related to tear film insufficiency has made little progress in recent years. Most current therapies aim to reduce drainage of tears from the eye, that is, punctual occlusion with cautery or plugs, or to replace insufficient aqueous production from the lacrimal gland with artificial tears. Each of these therapies reduces dry eye symptoms but each has significant drawbacks. For example, punctal plugs have poor retention rates; can migrate into the lacrimal system; predispose the eye to infection; and cause epiphora [86]. Punctal cautery can cause similar issues but is much more difficult to reverse with patient intolerance. Artificial tears are a more benign therapeutic option but the preservatives within them can be toxic to the cornea with frequent dosing [87]. This issue has been circumvented by the production of preservative-free preparations. While the ingredients have significantly changed, artificial tears were first described nearly 3,500 years ago and are unfortunately rapidly removed from the ocular surface [88]. It was not until the 1980s that natural or synthetic polymers were added to preparations increasing viscosity and retention time. Such compounds as 1% glycerin have shown prolonged benefit compared to propylene and polyethylene glycol are one such example [89]. Even with these advancements, artificial tears are only a temporary measure and do not provide important proteins produced by the lacrimal gland for ocular health as previously discussed and are short-lived. As such, artificial tears and punctal occlusion remain viable options for dry eye treatment but do not address the underlying lacrimal gland dysfunction.

Further advancements have been made with the introduction of cyclosporine. The compound was initially isolated from the fungus* Tolypocladium inflatum*, a potent inhibitor of T cell activity [90, 91]. Therapy with this medication has shown great effect by increasing goblet cell density and TGF-*β*. It has also been shown in mice to better reduce epithelial staining compared to prednisone in an inflammatory dry eye model [92]. However, this therapy is presumably most effective in an inflammatory dry eye minimizing its therapeutic use to these specific conditions. Additionally, poor patient compliance further reduces its widespread application due to ocular irritation and prolonged use necessary to see any appreciable benefit frustrating even the most compliant patients.

Multiple surgical attempts have been made to bypass the lacrimal gland altogether by transposing the parotid gland duct onto the lower conjunctiva, a technique developed in the 1950s [93]. This has shown great promise in dogs with keratoconjunctivitis sicca with a success rate as high as 92% [94], but the results are difficult to interpret in animals unable to voice complaints of dry eye or excessive tearing. We, as well as others, have all but abandoned the technique due to lack of any appreciable benefit, excessive tear secretion, and high rates atrophy of the gland following surgery [95]. Consequently, this technique is rarely used today except in animals due to inconsistent results and significant side effects.

## 4. Emerging Therapies for Dry Eye

There are several emerging modalities that have shown at least some promise in the basic science realm. These emerging therapies can be divided into two categories: exogenous compounds and gland regeneration/bypass. Unfortunately, many of these treatments are still in their infancy and have not made significant progress beyond the basic science realm.

Exogenous compounds are delivered to host tissue through either topical or oral routes. Many of these new agents directly inhibit proinflammatory cascades. The list of targets includes vascular cell adhesion inhibitors, immune modulators, and immune suppressants [96–98]. These inhibitors have shown efficacy in mouse models of inflammatory dry eye but have not been used in humans. One of these agents is delivered in an adenoviral vector, which would theoretically reduce the need for reapplication but raises concerns for inducing an innate immune response that could worsen inflammation [35, 98]. Moreover, immune suppression of the ocular surface could result in frequent infectious complications. The recently described nonimmune compound, pituitary adenylate cyclase-activating polypeptide-derived peptide, has been shown to promote corneal wound healing and lacrimal gland secretion in mice [99]. With the adenoviral vector as the exception, these compounds would likely be an improvement from artificial tears but would reduce ocular immunity. Patient compliance as seen with other topical eye medications would also be an issue. Consequently, the untested role of these topical therapies may be of some benefit in few select populations.

With increasing success in stem cell-based tissue regeneration, tissues and organs such as functioning photoreceptors and the liver can now be grown* in vitro* [100, 101]. Attempts of a bioengineered lacrimal gland have seen recent success in mouse models as well [102]. Stem cells are isolated using specific lacrimal cell markers, tissue grown* ex vivo*, and transplanted into the host resulting in increased tear production [102–104]. While promising, it remains to be seen whether these results can be reproduced in humans and provide a feasible, long lasting therapeutic option. There has also been some suggestion of using lacrimal gland xenografts as healthy tissue, but this theoretical idea remains untested [105]. Furthermore, these transplant models have not evaluated the effect of transplantation with ongoing diseases such as SS that may reduce graft transplantation rates and efficacy such as seen with a significantly higher rejection rate of herpes-infected corneas compared to noninflammatory corneal transplants [106]. While clinical promising and would address gland dysfunction and restore normal tear production, lacrimal gland regeneration or xenograph transplantation remains to be years from clinical use.

More recently, sublingual, labial, and submandibular glands have been transplanted into the subconjunctival space as an additional means to treat severe dry eyes due to underlying basal secretion of these glands that does not require innervation [107, 108]. The transplanted glands have shown a reduction in dry eye symptoms for at least five years and reduce the need for tear supplementation [109]. In addition, saliva contains many of the same contents as the lacrimal gland including secretory IgA but the two have not been specifically compared [110]. Unfortunately, the long-term efficacy beyond 5 years is currently unknown. Furthermore, the transplantation rate is at best 72% and requires a difficult microsurgery including vascular anastomosis of the gland to the temporal artery and vein [111]. As such, the surgery has not gained widespread use at this point in time. The transplants can also cause chronic inflammation exacerbating dry eye symptoms, microcystic epithelial edema, and epiphora in approximately 40% of patients within 3–6 months of surgery [112, 113]. While transplantation of these glands has shown great effect on dry eye and produce more natural tears than artificial instilled ones, the complicated surgery and risk of graft failure are staggering complications to overcome making them less than ideal.

Lastly, there is ongoing work on an implantable device to that stimulates the lacrimal nerve to increase tear production within the lacrimal gland and this small animal study has shown promising results [114]. However, it remains to be seen whether this method of hyperstimulating the lacrimal gland can and/or will overcome gland dysfunction and whether it becomes a feasible clinical treatment option. Furthermore, will this device be able to stimulate a diseased gland enough such as in SS to overcome the symptoms of dry eye?

In summary, there are several new modalities emerging for severe dry eye; however, many of these options remain unproven or require extensive, technically difficult microsurgery.

## 5. Conclusion

There are multiple disease entities that can affect the lacrimal gland and cause its dysfunction. Untreated pathologies and downstream effects of reduced production from the lacrimal gland can result in decompensation of the ocular surface and gross deterioration of visual acuity. The role of this gland cannot be overstated in ocular surface health and proper light refraction from the air-tear interface. Emerging therapies will hopefully alleviate the large dry eye burden worldwide by addressing the issue at its core, by attempting to regenerate a dysfunctional gland and/or controlling the proinflammatory state that ensues with severe dry eye. As such, new modalities and therapies need to be developed through collaborative/translational research to treat aqueous deficiency-related dry eye. It will be interesting to see if the untested, but promising, therapies discussed become viable treatment modalities in dry eye therapy beyond the temporary measures of ocular lubricants.

## Figures and Tables

**Table 1:** Causes of lacrimal gland dysfunction and their proposed pathological mechanism. Grosscategorization of the most common causes of lacrimal gland dysfunction based on underlying pathology most typical of the disease. HIV, human immunodeficiency virus; CMV, cytomegalovirus.

Pathological changes	Disease
Inflammatory/Oxidative Stress	Sjogren's syndrome
	IgG4-related disease
	Autoimmune Dacryadenitis
	Sarcoidosis
	Chronic graft-versus host
	Thyroid disease
	Orbital inflammatory pseudotumor
	Amyotrophic Lateral Sclerosis
	Diabetes
	Aging

Infectious	HIV
	CMV
	Hepatitis C

Atrophy	Aging
	Radiation

Toxicity	Radiation

Environmental	Smoking
	Video displays

Autonomic Dysfunction	Riley-Day syndrome

Idiopathic	
